# Bit type exerts an influence on self-controlled rein tension in unridden horses

**DOI:** 10.1038/s41598-020-59400-w

**Published:** 2020-02-12

**Authors:** Silvio Kau, Isabella Katharina Potz, Katharina Pospisil, Lina Sellke, Johannes Peter Schramel, Christian Peham

**Affiliations:** 1 Institute of Topographic Anatomy, Department for Pathobiology, University of Veterinary Medicine Vienna, Veterinärplatz 1, 1210 Vienna, Austria; 2 Movement Science Group, Equine Clinic, Department for Companion Animals and Horses, University of Veterinary Medicine Vienna, Veterinärplatz 1, 1210 Vienna, Austria

**Keywords:** Behavioural methods, Animal behaviour

## Abstract

Bit configuration and acting rein forces play a crucial role in oral health and comfort of ridden horses. Although it is a big animal welfare issue, dynamic response of horses to different bits has yet not been thoroughly investigated. This convenience sample experimental study describes a model to overcome the almost uncontrollable influence of riders on rein tension and evaluates self-controlled maximum side rein tension of ten sound horses randomly bitted with a double-jointed (DJS) and a version of a Mullen mouth snaffle-bit under unridden conditions. Horses were exercised at walk and trot on a horizontal treadmill wearing custom made force-sensing resistors (FSR) equipped to side reins. FSR were synchronized with a camera-based motion analysis system providing information on amplitudes and temporal occurrence of self-controlled maximum side rein tensile forces during different phases of separated motion cycles. The DJS exhibited larger side rein tension, indicating higher bit contact. Constant temporal occurrence of monophasic maxima at walk and biphasic maxima at trot could be observed in both bits. Within the limitations of this study, application of FSR linked to side reins in unridden horses may provide a promising tool when studying subjective response of horses to different bits.

## Introduction

Nowadays a huge variety of bit types made of different materials are used in equitation. In spite of that, very little is known about the mechanisms of action within the oral cavity and the subjective response of horses. Previous investigations reported that individual motion patterns and horse-rider interactions may be altered by the interdependence of bit configuration, acting rein forces, head and neck position of horses as well as the biodynamic laterality of horses and riders^1–7^.

Depending on the anatomy and functionality of different bit types, factors found to influence orodental health have been explored in several studies. Previous investigations identified bit position within the oral cavity and resulting effects on adjacent oral structures as dynamically depending on the applied rein tension^2^. Rein tension can be influenced by many intricate variables, such as the gait type and direction or type of movements^8–12^. However, rein tension-related response can vary among horses^13^, but a rider-independent assessment of the influence of different bits on horse-controlled rein tension is missing. In domestic sports and working horses, a high prevalence of periosteal bone spur formation of the inferior interalveolar margin and erosive lesions or fractures of lower second premolar dental hard tissues and canine teeth could be observed^14–17^. Likewise, oral soft tissue structures, such as the mucosal lining of the orolabial commissures or inferior interalveolar margins (bars) and tongue can get severely injured^3,16^. A study which determined the intraoral response of horses to different bits found that the examined bits (single-jointed, double-jointed and Myler comfort snaffle) did not affect pattern of intraoral behavior^13^. However, the bit type was shown to strongly influence location and severity of oral lesions^16^.

Ported curb bits most often cause severe damage to the bars whereas snaffle bits are more likely to cause buccal mucosal tears^16^. The double-jointed snaffle-bit (DJS) is supposed to be a comfortable bit by transferring more pressure more evenly to the tongue than a single jointed snaffle^18^. It was shown that the length of the central link of a DJS affects distribution of pressure to the tongue and bars^18,19^. A slightly curved Mullen mouth snaffle-bit (MMS) has a reduced risk of pinching the tongue by the central joint link, but can cause severe orodental damage if used improperly^19^. It is suggested, that different bit materials influence oral bit acceptance^20^, whereas changing inherent rein elasticity affects amplitude and transduction of tensile forces that occur in side reins under unridden conditions^21^ or when applied by the rider^22^. Regardless of the materials used, rein tension and aversive conflict behaviour appears to increase with decreasing rein length^21,23^. Dumbell *et al*.^24^ showed in a recent review that a growing amount of publications focuses on assessing human-horse interactions in equitation sports rather than studying the horse and human shares separately. This would be necessary for evidence-based evaluation of animal welfare concerns. It is assumed, that mainly the rider causes a variation of minimum rein tension which is considered as the baseline contact^21,25^. On the contrary the horse is believed to have a greater contribution on maximum rein tension values due to its dynamic head and neck movements^25^. Maximum rein tensile forces however, can be further amplified by the rider and hence may lead to increased pressure on oral tissues. As different bits and acting rein forces may compromise orodental health and hence are an animal welfare issue, it is important to know which maximum rein forces a horse is exerting and willing to accept without the uncontrollable influence of riders on rein tension^6,7,12,26^. To measure these forces we used side reins equipped with lightweight force sensors made of force-sensing resistors (FSR) on walking and trotting horses on a treadmill.

The goal of this study was to compare the self-controlled maximum side rein forces of horses randomly bitted with two different type of snaffle bits, one jointed and one rigid mouthpiece at different gaits using a full factorial experimental design. Hence the horses determine themselves the reins tension. We hypothesised that the tensile forces in the side reins change with different bits and that the forces are smaller with the MMS bit compared to the DJS bit. Furthermore, it was hypothesized that irrespective of the bit used at a given head position side reins tensile forces are higher at trot compared to walk due to differences in the head acceleration.

## Material and Methods

### Horses and instrumentation

Ten (*n* = 10) riding horses of different breed, sex, age (12.4 ± 5.3 years) and training experience were included in the study (Table 1). Three horses belong to the University of Veterinary Medicine Vienna (UVMV) whereas seven horses were provided by private owners. All horses were used to wear bits and bridles regularly. Prior to the study, all horses had experience with the DJS and one horse (no. 6) also had experience with the MMS. Two of the horses owned by the UVMV already had experience with the treadmill, though all horses participated in the pre-experimental treadmill training. The study was approved by the local ethics commission at the UVMV, Austria (under protocol number 10/05/97/2012). All methods were performed in accordance with the guidelines specified by the ethic commission to be based on the regulations of good scientific practice. Horse owners consented to the experimental set-up, data collection as well as the publication of results.

**Table 1:** Horses included to the study.

Horse ID	Breed	Sex	Age	Body weight [kg]	Height at the withers [cm]	Training experience
1	WBL*	Gelding	22	640	167	Experimental animal of the university**
2	WBL	Gelding	8	573	173	Dressage horse, 2^nd^ to 3^rd^ level
3	WBL	Gelding	18	481	168	Dressage horse, 3^rd^ level
4	Trotter	Gelding	12	577	165	Experimental animal of the university
5	WBL	Gelding	8	620	171	Dressage horse, 1^st^ to 2^nd^ level
6	WBL	Mare	16	590	165	Dressage horse, 2^nd^ to 3^rd^ level
7	WBL	Gelding	9	575	167	Leisure horse
8	WBL	Mare	15	487	152	Experimental animal of the university
9	Pinto	Gelding	5	490	158	Leisure horse
10	WBL	Gelding	11	570	164	Leisure horse

*WBL: Warmblood; **All experimental animals of the university used in this study were originally active sport horses used to bits and are now regularly ridden by students.

Initially, all horses were subjected to a clinical and subsequent specific lameness examination, which was carried out by an equine orthopaedic specialist and Diplomate of the ECVS a few days before the experiment at the equine clinic of the UVMV. Eligibility criteria required individuals to be free from apparent clinical signs and lameness when walking and trotting on hard and soft ground, circle and straight line. An orodental examination was performed by a Diplomate of the EVDC (eq) at the equine clinic of the UVMV. Horses were sedated by means of intravenous administration of detomidine hydrochloride (0.01 mgkg^−1^, Equidor, Richter Pharma) and butorphanol (0.025 mgkg^−1^, Alvegesic, Alvetra und Werfft). The oral cavity was thoroughly assessed using a full mouth speculum (Type Vienna) and oral endoscope (Karl Storz, Germany). All horses were free from orodental alterations like interalveolar bone spurs, mucosal ulcerations, incisival and/or cheek tooth malocclusions, missing teeth (excepting wolf teeth, as no horse had wolf teeth) or craniofacial deformities.

A few days prior to measurements, all horses were trained by IKP and KP to walk and trot on a horizontal high-speed treadmill (Mustang 2200, KAGRA AG, Switzerland). Every horse was exercised at ten minutes intervals for both walk and trot, twice a day for a period of five days according to Buchner *et al*.^27^ in compliance with recommendations of Bächi *et al*.^28^. In order to ensure motion pattern consistency, the individual optimum speed of movement for walk and trot was determined during the training phase^29^. On the last training day, the horses were equipped with the entire individually adjusted experimental setup except bits.

**Figure 1:**
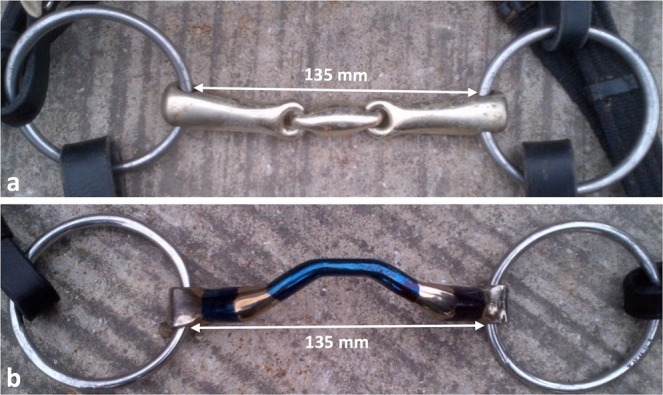
Snaffle-bit types used for cross-sectional investigations of self-controlled side rein tension. (**a**) Loose O-ring double-jointed snaffle-bit and (**b**). Loose O-ring Mullen mouth snaffle-bit. Arrows indicate mouthpiece widths.

For the purpose of analyzing bit-related self-controlled side rein contact a loose O-ring double-jointed (18 mm diameter, 135 mm width) and a loose O-ring Mullen mouth snaffle-bit (16 mm diameter, 135 mm width) (Fig. 1) were used in combination with a Hannoverian (drop-noseband) bridle. Bits and bridle straps were fitted according to Manfredi *et al*.^2^. The mouthpiece widths were >5 mm but ≤10 mm wider than the distance between the left and right orolabial commissures and the bridle was adjusted so that a small skin fold appeared at the commissures above the bit^2^. The tightness of the noseband was chosen allowing two fingers to fit underneath it in the nasal midline^30^.

**Figure 2:**
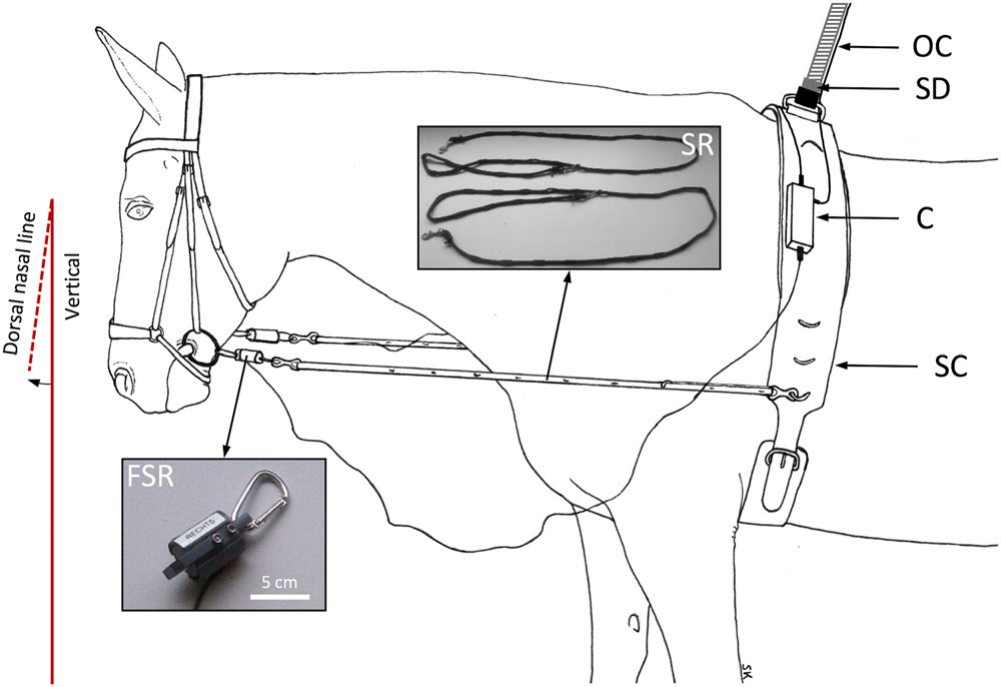
Schematic illustration of the experimental setting. Force-sensing resistors (FSR) interlink polycaprolactame side reins (SR) to the bit. Before caudal fixation of SR to the surcingle (SC), the length of the reins was individually adjusted so that the dorsal nasal line was slightly in front of the vertical (about 10 degrees). The force signals of the left and right FSR were passed through a connector (C) and following output cable (OC) to the analog digital converter. A mechanical safety device (SD) stops the treadmill in case of a fall.

Two force-sensing resistors (FSR) (RS Components, Corby, UK) mounted in a custom made housing having a mass of 27 grams were used to sense the rein forces. The left and the right sensor was rigidly connected to small elastic polycaprolactame side reins and attached to the bit ring with a lightweight snap hook^31^ (Fig. 2). Maximum load was 150N. Data were recorded at 120 Hz data rate and a resolution of 0.1N. FSR were calibrated prior to each measurement trial with masses between 500 g and 4000 g in steps of 500 g applied in an ascending and descending order. The calibration procedure was repeated three times. A calibration range of zero to 39.2N could be achieved. Outputs of both the left and right FSR were connected to an analog digital converter (ADC) which was synchronised with a kinematic acquisition system (Expert Vision System, Motion Analysis Corporation, CA, USA).

The caudal ends of the side reins with inserted FSR were attached to the to the lateral sides of a surcingle, which was tightly fastened around the girth line (Fig. 2). According to the dressage rules of the Fédération Equestre Internationale (FEI), the head position was adjusted such that the dorsal nasal line was slightly in front of the vertical^32^. This was achieved by adjusting the length and caudal position of both side reins in equal measure using removable lightweight snap hooks, fixed in regularly interspaced (10 cm distance) gaps of the reins (Fig. 2). During the measurements, the individual settings of the equipment were maintained for walk and trot. A vertically oriented automatic safety device was clipped to the top median ring of the surcingle to emergency stop the treadmill in case of a fall (Fig. 2).

Spheric retro-reflective skin-mounted markers (diameter of 1.9 mm, Motion Analysis Corporation, CA, USA), were attached to the lateral hoof wall of both forelimbs using cyanacrylat glue and adhesive fibre tape^29,33^. Markers were used to obtain kinematic data to assign side rein tensile forces to the phases of each motion cycle at walk and trot respectively.

### Data collection

Prior to each measurement horses were warmed-up on the treadmill for a minimum of one minute. Left (LSR) and right side rein (RSR) tension patterns were examined at walk and trot using both bits in a full factorial design, hence in every possible combination. The treadmill speed was set for each horse based on the previously determined optimum speed for walk and trot. Data were collected from several series of measurements (n = 3), each with three (n = 3) measurement trials for each combination of bit and gait type, resulting in a total of nine (n = 9) measurement trials per combination. In one trial, the motion cycle data and associated side rein tensile forces were recorded over a period of 10 seconds each after one minute of habituation. Recordings include data obtained from consistent and undisturbed walking or trotting sequences. Computer-assisted randomization was used to determine both the gait and order of used bits during measurements. Three-dimensional (3D) marker positions were recorded with ten infrared cameras placed at different heights (frame rate 120 Hz, resolution 1.3 Megapixels, Eagle Digital Camera, Motion Analysis Corporation, CA, USA). Prior to motion tracking, the system was calibrated using static (L-frame) and dynamic (wand) calibration^34^.

### Data analysis

**Figure 3:**
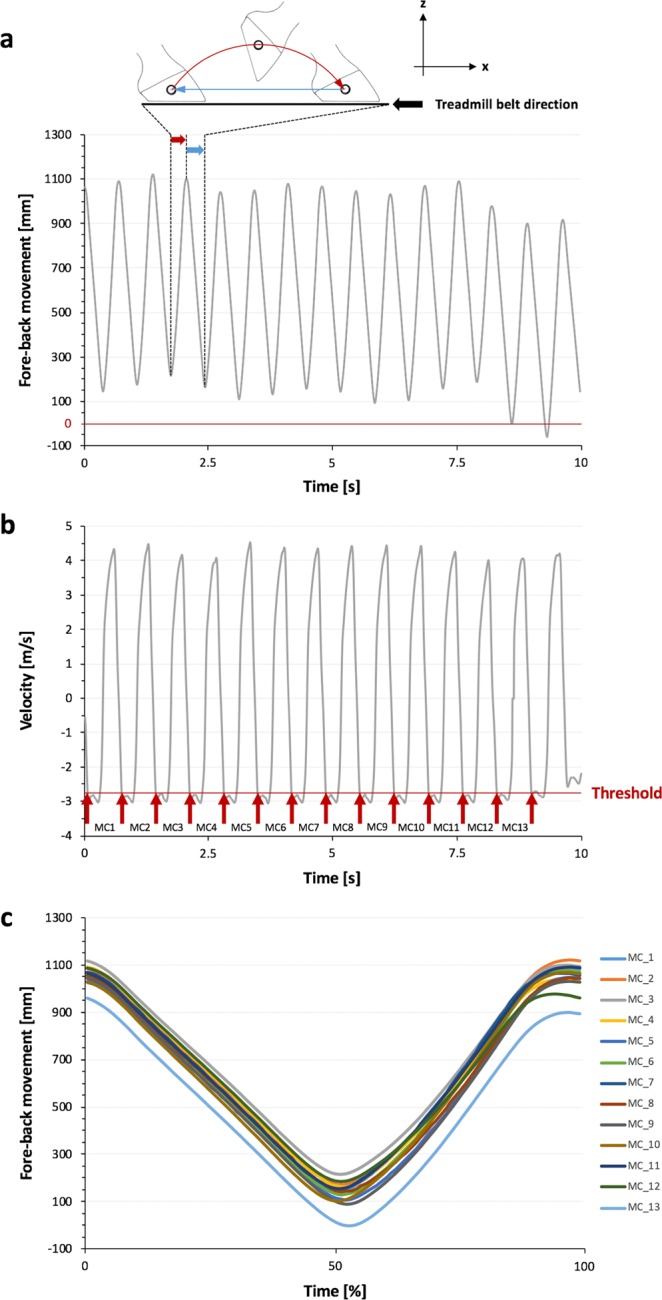
Decomposition of kinematic data and separation of motion cycles. (**a**) Horizontal fore-back movement (x-direction) of the retro-reflective reference marker on the left fore hoof. The system calibration defines origin of the 3D coordinate system. Negative values indicate movements beyond the origin. The time frame from 0 to 10 seconds defines one measurement trial. (**b**) The time derivative signal of the fore-back motion of the reference marker indicates horizontal velocity of the left fore hoof. Forward movement of the hoof has positive values, the backward movement negative. After setting a threshold level, motion cycles were separated (MC1-13). (**c**) Separated and resampled motion cycles (time normalised to 100%). The graphs show representative data of horse number 5 in trot.

First data (3D marker motions and side rein tension) were smoothed using a Butterworth low pass filter (third order; cut-off frequency 20 Hz; Expert Vision System, Motion Analysis Corporation, CA, USA). This procedure is a state of art filtering in motion analysis and other disciplines^21,35,36^. The sample rate of input sequences of side rein tension and kinematic data were normalized using the “Resample” function of the Signal Processing toolbox of MATLAB software (version 2013, MathWorks Inc., USA). This was to achieve time normalization of stride percentage. Thus, the duration of each motion cycle was 100 samples (=100% relative time of the motion cycles). Data recorded from FSR were normalized using the procedure described above, resulting in a stride split time (0 to 100%)^12^. Motion cycles were separated by using the kinematic data of the left fore hoof whereas speed of the horizontal movement of the hoof marker was considered as a reference^29,33^ (Fig. 3). The duration of each motion cycle was normalized to 100% starting with the landing of the left fore hoof at 0% (Fig. 3).

We determined the amplitude and the pattern of temporal occurrence (% of the motion cycle) of self-controlled maximum side rein tensile forces in the LSR and RSR for each motion cycle. Thereafter, we calculated the mean ± SD of these over available motion cycles and compared the data obtained by the use of both bits at walk and trot.

### Statistical analysis

Data analyses were performed using SPSS software (version 24.0, IBM Analytics, USA). Analytical results were graphically illustrated with Microsoft Excel (version 2016, Microsoft Corp., USA). The experiment was conducted using a repeated measures full factorial design (2 × 2 × 2) with gait type, bit type and the bilateral side reins as within-subject factors (fixed effects). None of the factors were treated as random effects. Quantitative data from repeated measures maximum side rein tensile forces and their temporal occurrence during separated motion cycles were the dependent variables. A three-way repeated measures analysis of variance (ANOVA) based on an univariate general linear model was used to statistically analyse the proportion of variation in the repeatedly measured dependent variables from all possible combinations of within-subject interaction terms including side reins (LSR and RSR), gait type (walk or trot) and bit type (DJS or MMS). The extracted quantitative data were tested for normality of residuals using a Kolmogorov-Smirnov test and for distortion of variances using Mauchly’s Test of Sphericity. The degree of reliance of observed significance levels was tested by confidence interval adjustment (95%) using a post hoc Bonferroni alpha correction procedure. For all statistical analyses, a P-value < 0.05 was considered as statistically significant and a P-value < 0.01 as highly significant respectively.

## Results

The Kolmogorov-Smirnov normality check revealed normal distribution of residuals from all possible fixed-factor interaction terms, P < 0.05. Outliers in the data (deviation ≥ 1.5 * SD from the mean) were all below the level 3 * SD indicating no extreme outliers to be present. Data exhibited no violations of sphericity. The tested data properties indicated applicability of the chosen statistical test procedure.

Participating horses had a mean body weight (±SD) of 560.3 ± 55.93 kg and mean height at the withers of 165 ± 6.11 cm. The optimum speed of movement with lowest motion pattern variation determined during the training session varied between 1.44–1.66 ms^−1^ at walk and 2.72–3.42 ms^−1^ at trot respectively. During one measurement trial, 7–10 walking and 12–15 trotting motion cycles were recorded for each bit. This in turn results in a total of 63–90 motion cycles observed for each bit at walk and 108–135 motion cycles at trot. Further analyses combine side rein tensile force values obtained from these motion cycles.

### Pattern of occurrence and temporal distribution of side rein tensile forces

**Figure 4:**
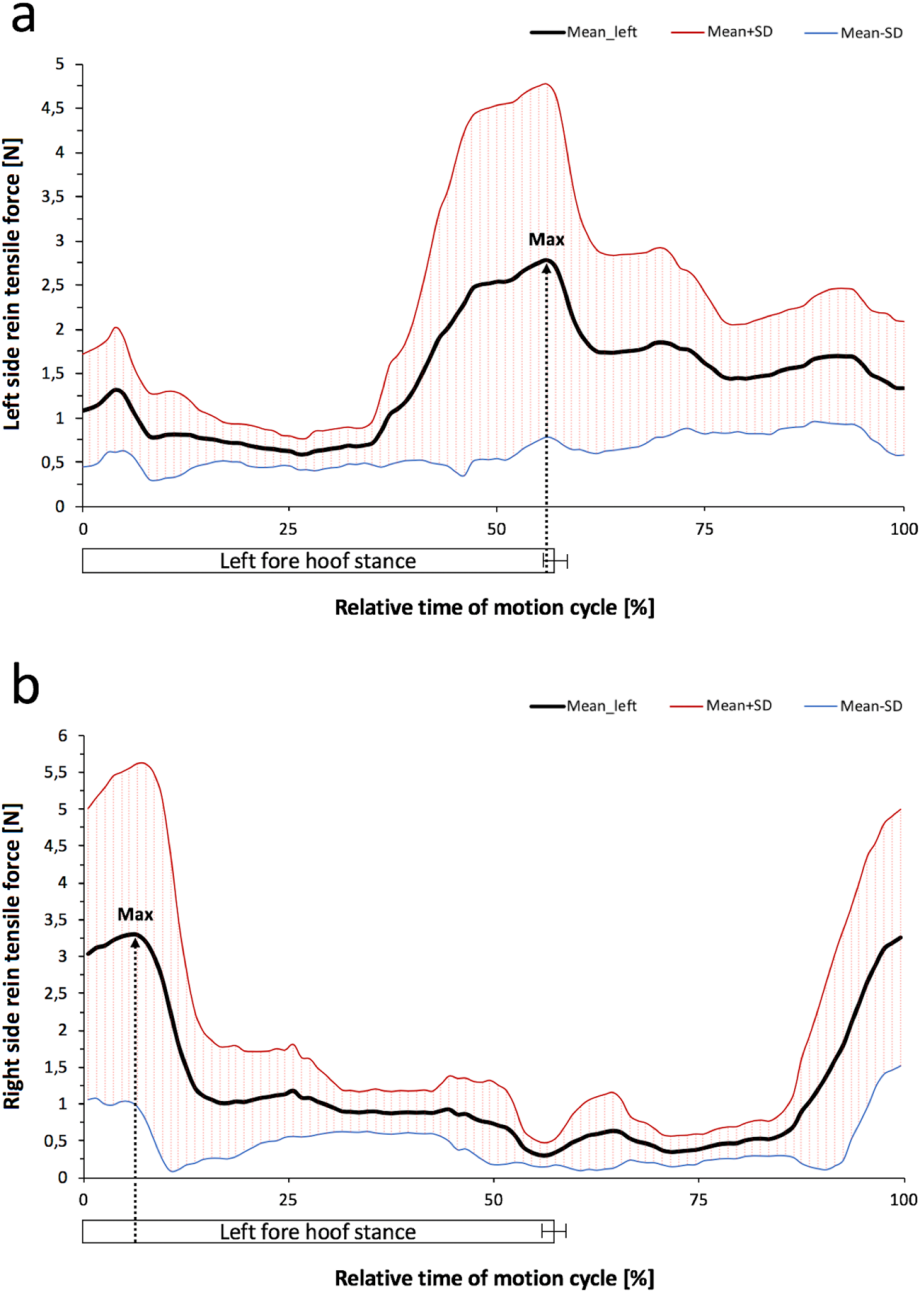
Course of the mean tensile force amplitude (±SD) in the left and right side rein at walk. Tensile force data were synchronised with separated and normalized motion cycles (0 to 100%), each beginning with the left fore hoof landing. The measurement trial (10 s) depicted in the graphs (a, b) comprises *n* = 8 motion cycles of horse no. 1 wearing a DJS. Mean duration (±SD) of the left fore hoof stance phase was 64.5 ± 1.3% of normalised motion cycles. Maximum side rein tensile forces (Max) appeared monophasic: (**a**) left side rein; Max in the end of the corresponding ipsilateral limb stance phase and (**b**) right side rein; Max shifted approximately by the half motion cycle.

**Figure 5:**
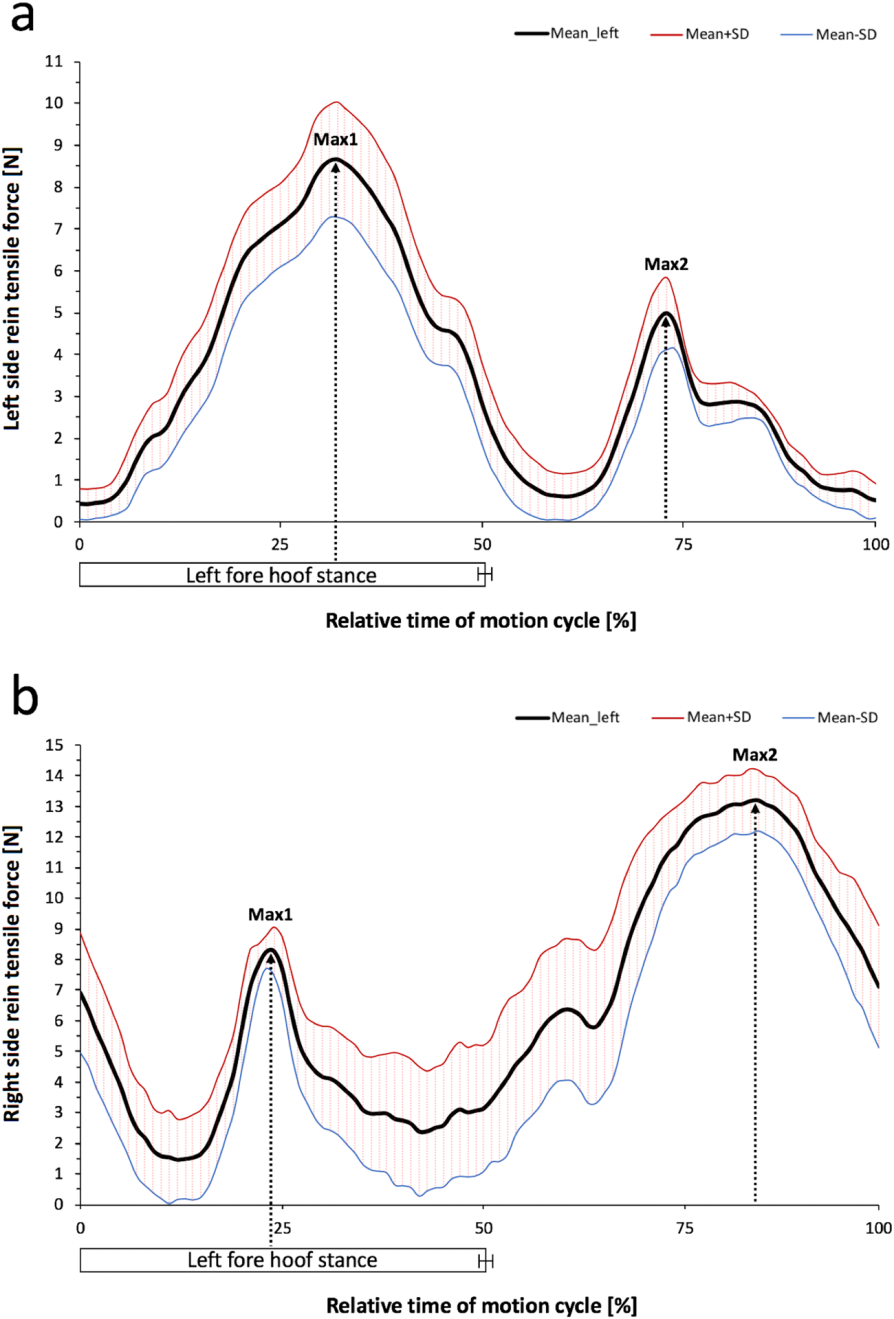
Course of the mean tensile force amplitude (±SD) in the left and right side rein at trot. Tensile force data were synchronised with separated and normalized motion cycles (0 to 100%), each beginning with the left fore hoof landing. The measurement trial (10 s) depicted in the graphs (a, b) comprises *n* = 13 motion cycles of horse no. 2 wearing a MMS. Mean duration (±SD) of the left fore hoof stance phase was 50.2 ± 0.7% of normalised motion cycles. Maximum side rein tensile forces (Max1, Max2) appeared biphasic: (**a**) left side rein; Max1 over half of the corresponding ipsilateral limb stance phase; Max2 at middle of the contralateral limb stance phase and (**b**) right side rein; Reversed difference in magnitude of Max1 and Max2.

The temporal occurrence of mean maximum side rein tensile forces differed between the two gaits investigated. Regardless of the bit used, force maxima always appeared monophasic at walk and biphasic at trot in all horses. The Figs. 4 and 5 show representative force curves generated from data (mean ± SD) of several motion cycles of one horse. At walk, all maxima occurred at the end of the corresponding ipsilateral fore hoof stance phase (Fig. 4). This coincided with the lifting of the left fore hoof (beginning of the second half of the motion cycle) for Max in the LSR and with the landing of the left fore hoof (in the first half of the motion cycle) for Max in the RSR (Fig. 4). Additional normalisation of motion cycles based on the left and right fore hoof revealed no overall difference in the mean time of occurrence of monophasic tensile force maxima between the bits in both the LSR (60.7 ± 26%) and RSR (62.0 ± 21%).

**Figure 6:**
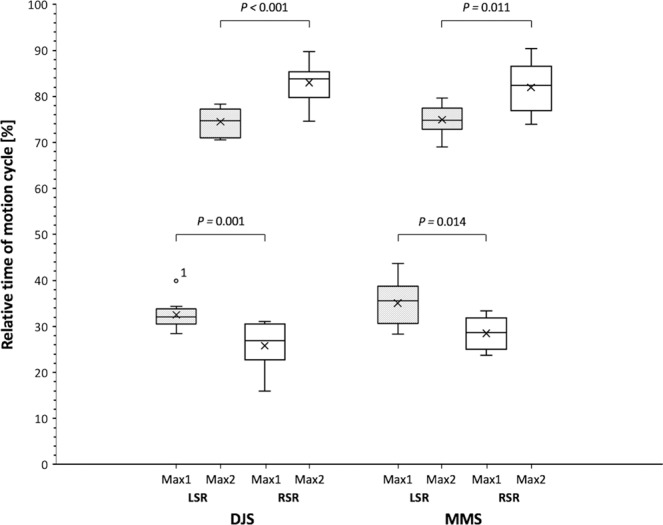
Overall chronology of biphasic maximum tensile forces (mean ± SD) comparing left (LSR) and right side reins (RSR) and a double-jointed (DJS) and Mullen mouth snaffle-bit (MMS) during normalized motion cycles (0 to 100%) at trot. Regardless of the bit used, the first force maximum (Max1) constantly appear in the first (0 to 50%) and Max2 in the second half (51 to 100%) of each motion cycle. Comparing both bits, corresponding force maxima constantly occur in the same time frame. In both bits, Max1 occurs slightly later in the LSR compared to the RSR and contrarily, Max2 occurs slightly later in the RSR. P-values are displayed in the graph. P-values are from an ANOVA analysis; A P-value ≤ 0.05 was considered significant; *n* = 10 horses. Box-plot elements: Box spans the interquartile range 25 to 75% (quartile 1, 3); vertical line (median); whiskers extend to highest and lowest values observed; outlier of LSR Max1 of horse no. 10 is displayed as dot.

Similar for both bits and both side reins, at trot the first tensile force maximum (Max1) occurred always during the first half (0–50%) and the second force maximum (Max2) during the second half (51–100%) of all motion cycles (*P* < 0.001) (Figs. 5, 6). Max1 in both side reins appeared around the middle of the left fore hoof stance phase (between 20–40% of the normalized motion cycles) usually starting with Max1 in the RSR. Max2 in both side reins appeared between 70–90% of the normalized motion cycles which corresponds to mid stance of the contralateral right fore hoof. Max2 in the LSR appeared first in most cases (Figs. 5, 6).

### Amplitude of maximum side rein tensile forces at walk

**Figure 7:**
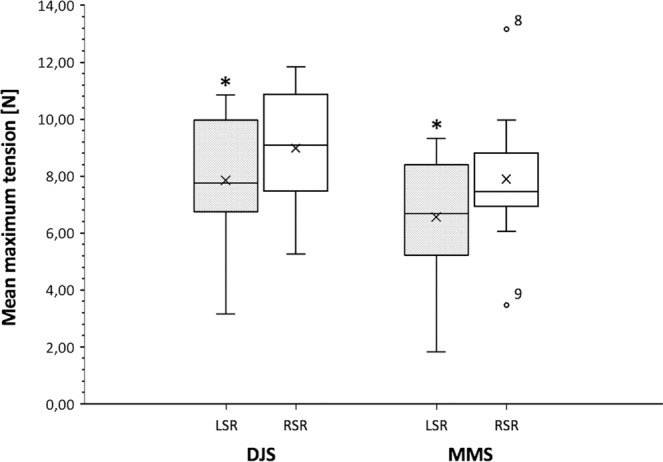
Overall maximum tensile forces (mean ± SD) in side reins at walk. There was no difference of occurring maximum forces between the left (LSR) and right side rein (RSR) neither in the double-jointed (DJS) nor Mullen mouth snaffle-bit (MMS). Comparing both bits, mean maximum tensile forces appeared significantly higher in the LSR when using the DJS. Statistical significance is indicated by asterisks (*) *P* = 0.043. However, there was no statistical significant difference in the RSR (*P* = 0.315). P-values are from an ANOVA analysis; A P-value ≤ 0.05 was considered significant; *n* = 10 horses. Box-plot elements: Boxes span the interquartile range 25 to 75% (quartile 1, 3); vertical line (median); whiskers extend to highest and lowest values observed; outliers of occurring forces in the RSR of horse no. 8 and 9 are displayed as dots.

The comparison of mean maximum tensile force values revealed no significant difference between the LSR and RSR in both bits. Mean maximum side rein tensile forces of the study group were higher in the DJS. The mean difference ± SD for the LSR was 1.3 ± 1.7N and 1.1 ± 3.2N for the RSR. A significant difference, however, was only shown for the LSR (Fig. 7). Mean values of all measurements of individual horses are listed in Table 2.

**Table 2:** Monophasic maximum tensile forces in the left (LSR) and right side rein (RSR) using a double-jointed (DJS) or Mullen mouth snaffle-bit (MMS) at walk.

Horse ID	Bit type			
	DJS		MMS	
	LSR	RSR	LSR	RSR
1	3.16	6.01	1.83	7.28
2	7.82	9.57	9.32	9.98
3	5.91	5.27	5.50	7.57
4	7.03	9.94	6.10	7.33
5	7.14	7.96	4.34	6.82
6	10.84	10.59	8.39	6.07
7	10.34	11.71	6.27	9.00
8	9.83	8.31	7.10	13.15
9	7.69	8.63	8.47	3.46
10	8.78	11.83	8.31	8.26
Mean	7.85	8.98	6.56	7.89
±SD	2.28	2.20	2.28	2.55

Mean values of all measurements of different horses (n = 10) and mean values ± standard deviation of the study group are indicated.

### Amplitude of maximum side rein tensile forces at trot

**Figure 8:**
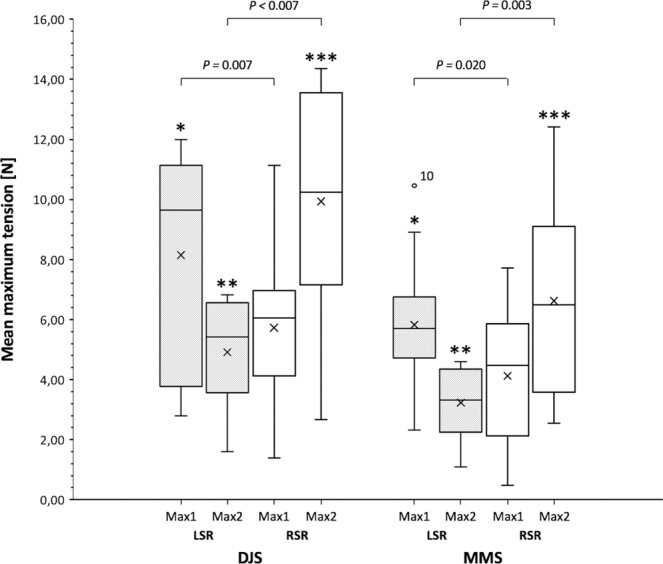
Overall maximum tensile forces (mean ± SD) in side reins at trot. Notice the significant difference of occurring maximum forces (Max1, Max2) between the left (LSR) and right side rein (RSR) in both bits, the double-jointed (DJS) and Mullen mouth snaffle-bit (MMS). P-values are displayed in the graph. Max 1 and Max2 of the LSR and Max2 of the RSR differ between the two bits, with larger tensile forces in the DJS. Statistical significance is indicated by asterisks (*) *P* = 0.022, (**) *P* = 0.011 and (***) *P* = 0.006. However, higher values of Max1 in the RSR of the DJS were not approved to be significantly higher than those in the RSR of the MMS (*P* = 0.178). The pattern of amplitudes is the same for both bits, with Max1 larger in the LSR and Max2 in the RSR. P-values are from an ANOVA analysis; A P-value ≤ 0.05 was considered significant; *n* = 10 horses. Box-plot elements: Boxes span the interquartile range 25 to 75% (quartile 1, 3); vertical line (median); whiskers extend to highest and lowest values observed; outlier of LSR Max1 of horse no. 10 in the MMS is displayed as dot.

Biphasic occurring mean maximum tensile forces significantly differed between the DJS and MMS and between the LSR and RSR in individual bits. The mean amplitude of Max1 of the study group was higher in the LSR and Max2 in the RSR. The mean difference ± SD for maxima in the LSR was 2.4 ± 2.8N and 3.6 ± 3.1N for the RSR. Comparison of maximum forces between the bits revealed, that Max1 and Max2 in the LSR and Max2 in the RSR differed significantly, with larger maximum tensile forces emerging in the DJS (Fig. 8, Table 3).

**Table 3:** Biphasic maximum tensile forces (Max1, Max2) in the left (LSR) and right side rein (RSR) using a double-jointed (DJS) or Mullen mouth snaffle-bit (MMS) at trot.

Horse ID	Bit type							
	DJS				MMS			
	LSR		RSR		LSR		RSR	
	Max1	Max2	Max1	Max2	Max1	Max2	Max1	Max2
1	3.68	2.30	1.40	2.67	2.39	1.71	1.26	2.54
2	9.38	4.29	6.08	12.51	8.91	4.35	7.71	12.41
3	2.79	3.99	4.12	8.59	5.27	4.56	6.31	4.45
4	4.06	3.41	4.15	6.67	4.39	3.21	4.73	7.82
5	11.14	6.67	11.14	14.18	5.11	2.20	2.05	6.28
6	9.91	5.62	5.40	9.98	6.67	2.80	5.97	5.29
7	11.34	6.22	7.13	14.35	7.00	4.06	5.55	9.53
8	11.08	6.83	6.49	13.74	6.35	4.59	4.17	6.71
9	10.24	6.71	7.79	9.19	4.83	2.37	0.47	3.17
10	12.00	6.08	6.44	12.99	10.46	4.34	4.66	11.03
Mean	8.56	5.21	6.01	10.49	6.14	3.42	4.29	6.92
±SD	3.58	1.60	2.58	3.81	2.31	1.09	2.34	3.28

Mean values of all measurements of different horses (*n* = 10) and mean values ± standard deviation of the cohort are indicated.

## Discussion

The present study was designed to determine the influence of two different snaffle bits on self-controlled side rein tensile forces in unridden horses at walk and trot on a treadmill. Our investigations revealed mean maximum tensile force amplitudes being lower in the MMS compared to the DJS in both, walk and trot. The amplitude of tensile forces was close in both gaits and occurred monophasic at walk and biphasic at trot. Despite the fact that different bits may have a significant impact on animal welfare, very little is known about their manifold influencing variables^5^. The measurement of effective pressure evoked by different bits on oral tissues of distinct elasticity, however, remains a major technical challenge highlighting the need for alternative approaches to measure subjective response of horses.

### Treadmill locomotion and pre-experimental training phase

It is supposed, that symmetry and amplitude of rein tension forces differ highly in the course of equitation movements but should remain rather constant when horses are ridden on a straight line^11^. Rein tension pattern can vary through whole riding sessions and may be affected by horses and riders respectively^37^. The major variation of minimum rein tension forces which is considered as the baseline contact is assumed to be caused by the rider^21,25^. In order to overcome the almost uncontrollable influence of the rider on rein tension and to standardize conditions, the study setup used unridden horses exercised on a horizontal high-speed treadmill instead of overground locomotion. Since a treadmill can be operated at constant speed and straight direction even over longer distances investigations are probably less biased compared to overground conditions. However, it is important to consider kinematic and biomechanical differences between treadmill and overground locomotion as well as the flooring type when comparing results of studies using different setups^38^. For rein force studies, it was recommended to consider further influencing variables such as the gait type, rider position, movements performed, and educational level as well as laterality of both the rider and the horse^10^.

Research on ridden horses demonstrated that rein tension frequently differs between the left and right rein. Several studies have shown that this can be strongly influenced by body asymmetries as well as dynamic laterality of both the rider and the horse^6,7,26^. There were no data available about laterality of the horses included in this study. However, our results revealed no significant difference of LSR and RSR in both bits. Nevertheless, 80% of the horses showed higher forces occurring in the RSR. This was probably caused by the experimental setting where horses tended to orientate themselves on the handling person standing to the left side of the treadmill during examinations.

Horses should get accommodated to treadmill exercise prior to investigations as motion pattern occurs unsteady when using a treadmill the first time and at the beginning of any exercise^27,28^. This may influence results of studies investigating variations in rein tension. Training horses twice a day for five minutes at walk and trot, leads to kinematic motion pattern consistency at trot but variables can still appear altered at walk. Thus longer training periods were recommended^27^. For this study we trained horses ten minutes twice a day during five days and used a one minute warmup prior to measurement. Motion pattern consistency at trot on a treadmill highly depends on the individual speed of movement^29^. Hence we determined the optimum speed of movement at walk and trot during the training phase.

### Snaffle-bits and temporal occurrence of tensile force maxima

Both the DJS and MMS, are considered to be rather comfortable bits when used correctly. A slightly curved MMS is assumed to distribute rein forces evenly to the tongue without mediating significant pressure to the bars. By contrast a DJS is supposed to transfers pressure to the tongue and the bars^18,19^. However, there is little evidence-based data of the mechanism of these bit types. The DJS and MMS used in our study, had loose O-shaped bit rings which are suggested to provide a direct force transmission without lever action^19^. Contrary, a recent study demonstrated that such loose O-ring bits can also cause mild poll pressure by pulley-like transfer of approximately 20% of acting rein forces^39^. The authors assume that within the implemented setup residual leverage may not have influenced amplitude or difference in self-controlled side rein tension in both bits. The results suggest that the DJS may cause a similar oral sensory than the MMS despite a higher side rein tension.

The DJS and MMS used were individually inspected to ensure an appropriate seat within the previously described fitting parameters^2^. The bits differed 2 mm in diameter (DJS: 18 mm, MMS: 16 mm) and had a width of 135 mm. Thick mouthpieces may cause oral discomfort in horses with a small upper-lower jaw distance. Large breed (Warmblood) horses have a distance between upper and lower jaw bars between 25–44 mm^40^. Hence it is unlikely that the difference in thickness had a significant influence on amplituede and pattern of side rein tensile forces.

The side rein tension pattern in our study consistently exhibited monophasic force maxima at walk but biphasic force maxima at trot. The latter is broadly consistent with earlier findings in ridden and unridden horses^6,12,21,31^. A previous investigation showed that maximum forces may also be biphasic at walk, which is probably depending on the riders influence^12^. The monophasic tensile force maxima at walk largely appeared in the end of the corresponding ipsilateral fore hoof stance phase in both side reins. To the best of the authors´ knowledge, there are no reference rein tension data for unridden horses at walk. Egenvall *et al*.^12^ reported that in ridden horses maximum rein tension typically appears at hindlimb stance phase. The shift of temporal maximum force occurrence is most likely due to the influence of movement type and the rider as well as laterality effects. Rein tension at trot exhibited maximum forces in the respective second half of each diagonal stance phase which is in line with previous findings in both ridden and unridden horses^12,21^. It was shown that the two force maxima at trot occur at 10–30% and 60–80% of the stride in ridden horses^12^. Biphasic maximum tensile forces in our study appeared within the same time period at 20–40% and 70–90% of individual motion cycles. Interestingly, the biphasic force maxima at trot significantly occurred time-lagged (Max1 right – Max1 left – Max2 left – Max2 right). If the laterality of the horses had significantly influenced this pattern, then both maxima of one side rein should have occurred after the maxima of the contralateral rein. We therefore assume that the forward and backward movement of the forelimbs and the consequent displacement of the surcingle could have influenced the specific sequence pattern. This should be considered in future studies. Another study reported that in horses ridden at trot, however, the same head/neck position as chosen for our investigations revealed highest rein forces during swing phase or mid-stance phase^6^. This result in turn suggests that the rein tensile force maxima in horses either ridden or unridden can occur at different stages of the motioncycle.

### Gait types and amplitudes of tensile force maxima

One unanticipated finding in the current study was that mean maximum tensile forces for both the DJS and MMS were close at walk and trot. Several rein tension studies using ridden horses showed that tensile forces at walk are lower than occurring at trot^8,10,12,26,37^. A recent study investigated the rein tensions at walk, trot and canter in ridden and unridden horses. In ridden horses tensile forces had lower amplitudes at walk compared to trot and canter whereas without a rider the differences were smaller between gaits and had lower amplitudes^41^.

In our study maximum forces rarely exceeded values up to 22.5N at walk and 20.0N at trot. A recent literature review showed that experiments performed on ridden horses exhibited markedly higher rein tension values for both walk and trot^24^. Maximum force values under ridden conditions were demonstrated peaking up to 50.0 N^8,11,26,42^ or even more^37^. These findings suggest that the movement of the rider, due to transmitted forces from the horseback, may result in increased tension on the reins even if the rider strives for an independent steady hand and rein position^10,43^. In addition, rein material was shown to affect force transduction under ridden conditions^22^. Another reason for higher maximum values on ridden horses could be the direction of the reins, which is diagonally upwards under ridden and almost horizontal under unridden conditions, as described in Piccolo and Kienapfel^41^.

Furthermore, it was shown that horses worked in a less restrained way were more likely to show lower tensile forces in the reins^12^. A higher head position shortens the stride length at walk whereas at trot the head position seems not to have a significant kinematic impact^44^. We did not change the head/neck positions between measurement trials in the current study. A change in the head position would have been accompanied by a change in the length of the side reins and possibly would have influenced the tensile force amplitude^21^. Subjective observations revealed that quiet horses stabilized their neck on the side reins at walk, whereas more alert horses sometimes pushed against the reins. Young bit and bridle naive horses bitted with a DJS for any purpose, learned to avoid rein tension at different rein lengths^23^. The horses in this study showed greater discomfort the shorter the reins were. Self-controlled rein forces on day three were almost half as high as on the first day which may suggest accommodation effects. In short reins peak forces up to 38.0 ± 1.6 N were observed^23^. Overall maxima in our study were far below for both walk (DJS: 8.98 ± 2.2 N; MMS: 7.89 ± 2.55 N) and trot (DJS: 10.48 ± 3.81 N; MMS: 6.92 ± 3.28 N). Compared with the DJS, mean maximum tensile forces showed lower amplitudes in the MMS. Despite the significant difference in force maxima between the two bits, the maximum amplitudes were not far apart. However, results of Christensen *et al*.^23^ differ from the findings presented here. This may be explained by differences in study setups. We did not used positive operant conditioning and implemented a realistic dynamic movement situation. Interestingly horse 6, which was the only one that was in prior touch with the MMS, exhibited a greater mean difference between the bits compared to most other horses indicating some degree of response individuality. Treadmill training sessions were performed without bits. So habituation effects in the course of the measurements were still possible. According to Clayton *et al*.^21^, we used a more neutral length of the small elastic side reins and therefore less restrained head/neck position. They showed decreasing rein tension with increasing length and elasticity of side reins under unridden conditions and found maximum tensile forces at neutral head/neck position reaching 9.76 ± 0.33 N in stiff-, 7.29 ± 0.32 N in compliant- and 11.54 ± 0.32 N in non-elastic side reins^21^. These findings are most consistent with the force amplitudes found in our study.

### Study limitations

The investigations of the present study are confined to a small number of horses (*n* = 10) and hence results need to be interpreted with caution. The horses of this convenience sample experimental study partly had a different training status. Furthermore, most of the horses were not familiar with the MMS prior to the study and might have responded more confidently with the DJS. Another limitation is that although we have checked for the correct bit fit, the same bits have been used in all participating horses and were not individually adapted.

## Conclusion

Side rein tensile forces in unridden horses do not correspond to the forces certainly acting in the oral cavity during riding, but they may provide information of bit induced oral discomfort. The force maxima however, were lower than those reported in earlier studies on ridden horses. Our results suggest that the side rein tension force caused by the horse is much lower than during riding and may vary with the bit type. Bringing rein tensions in ridden horses down to the self-induced level is of great importance and an animal welfare issue. The experimental setting suggests a valuable method to objectively investigate the individual response of horses to different bits.

## Data Availability

The authors declare that all data supporting the findings of this study are available within the article or from the corresponding author upon reasonable request.
